# The European livestock resistome

**DOI:** 10.1128/msystems.01328-23

**Published:** 2024-03-19

**Authors:** Patrick Munk, Dongsheng Yang, Timo Röder, Leonie Maier, Thomas Nordahl Petersen, Ana Sofia Ribeiro Duarte, Philip T. L. C. Clausen, Christian Brinch, Liese Van Gompel, Roosmarijn Luiken, Jaap A. Wagenaar, Heike Schmitt, Dick J. J. Heederik, Dik J. Mevius, Lidwien A. M. Smit, EFFORT Consortium, Haitske Graveland, Alex Bossers, Frank M. Aarestrup

**Affiliations:** 1 National Food Institute, Technical University of Denmark, Lyngby, Denmark; 2 Institute for Risk Assessment Sciences, Faculty of Veterinary Medicine, Utrecht University, The Netherlands, Utrecht; 3 School of Biological Sciences, University of Edinburgh, Max Born Crescent, Edinburgh, United Kingdom; 4 Department of Infectious Diseases and Immunology, Faculty of Veterinary Medicine, Utrecht University, The Netherlands, Utrecht; 5 Wageningen Bioveterinary Research, Wageningen University & Research, Lelystad, The Netherlands; Kobenhavns Universitet, Copenhagen, Denmark

**Keywords:** resistome, livestock, metagenomics, antimicrobial resistance, diversity

## Abstract

Metagenomic sequencing has proven to be a powerful tool in the monitoring of antimicrobial resistance (AMR). Here, we provide a comparative analysis of the resistome from pigs, poultry, veal calves, turkey, and rainbow trout, for a total of 538 herds across nine European countries. We calculated the effects of per-farm management practices and antimicrobial usage (AMU) on the resistome in pigs, broilers, and veal calves. We also provide an in-depth study of the associations between bacterial diversity, resistome diversity, and AMR abundances as well as co-occurrence analysis of bacterial taxa and antimicrobial resistance genes (ARGs) and the universality of the latter. The resistomes of veal calves and pigs clustered together, as did those of avian origin, while the rainbow trout resistome was different. Moreover, we identified clear core resistomes for each specific food-producing animal species. We identified positive associations between bacterial alpha diversity and both resistome alpha diversity and abundance. Network analyses revealed very few taxa–ARG associations in pigs but a large number for the avian species. Using updated reference databases and optimized bioinformatics, previously reported significant associations between AMU, biosecurity, and AMR in pig and poultry farms were validated. AMU is an important driver for AMR; however, our integrated analyses suggest that factors contributing to increased bacterial diversity might also be associated with higher AMR load. We also found that dispersal limitations of ARGs are shaping livestock resistomes, and future efforts to fight AMR should continue to emphasize biosecurity measures.

**IMPORTANCE:**

Understanding the occurrence, diversity, and drivers for antimicrobial resistance (AMR) is important to focus future control efforts. So far, almost all attempts to limit AMR in livestock have addressed antimicrobial consumption. We here performed an integrated analysis of the resistomes of five important farmed animal populations across Europe finding that the resistome and AMR levels are also shaped by factors related to bacterial diversity, as well as dispersal limitations. Thus, future studies and interventions aimed at reducing AMR should not only address antimicrobial usage but also consider other epidemiological and ecological factors.

## INTRODUCTION

Treatment failure due to antimicrobial resistance (AMR) is considered one of the largest threats to human and animal health (1–6). Multiple studies have shown that AMR bacteria and, in some cases, antimicrobial resistance genes (ARGs) can be transferred between livestock and humans (7, 8). However, despite decades of research, it remains difficult to determine the size and importance of individual transmission pathways, partly due to the complex epidemiology of AMR. ARGs are in many cases not confined to a single bacterial species but may transmit between different gut commensal species prior to emerging in human pathogenic bacteria. Most studies conducted to date have focused on single indicator species or specific human pathogens and largely missed the emergence and dispersal of AMR in the normal gut microbiota.

With the developments in genomic sequencing, it has become technically and economically feasible to characterize any animal microbiome and their associated ARG reservoirs, the resistomes. It has also been documented that metagenomic methods offer improvements over traditional phenotypic AMR surveillance of indicator species by more closely capturing increases in AMR due to antimicrobial usage (AMU) than indicator species can (9–11).

In the Ecology from Farm to Fork Of microbial drug Resistance and Transmission (EFFORT) project, we previously analyzed pooled fecal samples from 181 pig herds and 178 broiler flocks in nine European countries using shotgun metagenomic sequencing (12). We found higher AMR loads in pigs, whereas broiler resistomes were more diverse. In addition, we identified a number of core ARGs strongly associated with the two livestock populations. This is highly important for studies trying to perform source attribution and elucidate the relative transmission of AMR between reservoirs (13). We have also shown that the farm-specific use of antimicrobial agents was significantly associated with AMR but only explains a limited amount of variation in AMR load (14, 15).

In the current study, we further expanded on the integrated analysis of European livestock resistomes (12) by including samples from veal calves, turkeys, and rainbow trout, using updated bioinformatic and downstream analyses of the combined results of all five animal categories, providing a comprehensive overview of potential human occupational and food-related exposure to livestock-related AMR within Europe. Moreover, we investigated the importance of AMU and the associations between bacterial and resistome diversity and abundance. Finally, we analyzed the association of overlap and dissimilarity in resistomes to answer whether the ARG patterns are best explained by differential selection or dispersal limitations.

## MATERIALS AND METHODS

Here, we present a combined analysis of the resistomes from pigs, broilers, veal calves, turkeys, and fish from different European countries (Data S1 and S2). Comparative analyses of the resistomes of broilers and pigs have previously been published (12), as have source attribution analyses of resistomes from pigs, broilers, veal calves, and turkeys (13) and risk factor analyses for resistomes in pigs (14), broilers (15), and turkeys (16), individually. A combined analyses have, however, never been performed. Also, compared to previous studies, we updated the workflow to incorporate faster DNA alignment methods (17), filtered lower-coverage ARGs to minimize spurious signal, and analyzed the data sets using compositionally correct methods as detailed below (18). Because each data set has a fixed number of reads determined by the sequencing instrument, the number of reads assigned to each feature is not independent of each other and should be treated as part of a composition (18).

The centered log ratio (CLR), additive log ratio (ALR), and interquartile log ratio (IQLR) are examples of methods originally proposed by John Aitchison to analyze compositions and are recommended for analyzing microbiomes, which are compositional (18, 19).

### Sampling procedure

Livestock from conventional pig, broiler, and turkey farms in nine European countries was sampled as previously described (12, 16). In addition, for this study, we included industrial veal calf (white/rosé), turkey, and rainbow trout productions from three countries each. A breakdown of the 538 sampled herds by livestock species and country is presented in Data S1. The protocols for farm selection and sample collection are more thoroughly described in the supplement. It is important to note that convenience and accessibility were allowed to influence herd inclusion choice, so the sampled herds could differ from average national conditions.

After 25 randomly distributed fecal samples (pigs, broilers, veal calves, and turkeys) or intestine contents (rainbow trout) were collected at each farm close to the date of slaughter (date of sale for freshly caught trout), pooled samples representing each herd were established: the 25 individual samples from each herd were pooled, with individual samples all contributing equal mass. The pooled samples were stored at −80°C locally and sent in batches on dry ice to the Technical University of Denmark (DTU).

### DNA extraction and sequencing

Procedures for DNA extraction and sequencing of samples from veal calves, turkeys, and trout were designed to be as similar as possible to the ones used by Munk et al. (12) to evaluate the pig and broiler resistomes.

The trout sampling strategy deviated slightly in the following manner. At each farm, 25 live rainbow trout were collected close to their sale date. After euthanasia, the gastro-intestinal tracts were removed and frozen at −80°C and shipped for processing at DTU on dry ice. The freezing procedure made it difficult to separate fish intestines and fecal contents. Therefore, for each fish farm, 0.2 g of the intestines for each of the 25 trout was pooled, similar to the procedure of the other animal species.

After pooled veal calf and turkey samples were obtained, DNA extraction was performed as for the previous pig and broiler samples. Briefly, we extracted DNA from herd-level fecal pools using a previously published bead-beating-optimized standard operating protocol based on the QIAamp Fast DNA Stool Mini Kit (51604, Qiagen) (20).

Two batches of DNA extracted from calf and turkey pooled fecal samples were shipped on dry ice to Admera Health (South Plainfield, NJ, USA) where library preparation and shotgun metagenomic sequencing were performed. Briefly, after DNA fragmentation (Covaris LE220), sequencing libraries were prepared and multiplexed using the KAPA HyperPrep kit (Kapa Biosystems). While libraries corresponding to veal calf samples were prepared without PCR (same as for pigs), the library preparation for turkey samples involved limited PCR amplification (same as for broilers). The generated libraries were sequenced on the NovaSeq 6000 platform (Illumina), using a 2 × 150-bp paired-end (PE) read approach.

DNA obtained from rainbow trout samples was prepared for sequencing using the Nextera XT library preparation kit (Illumina, FC-131-1024) and sequenced in batches of six samples on the NextSeq 550 platform (Illumina) internally at DTU.

### Processing and alignment of reads

Biological replicate samples were collected at eight farms: two pig farms were sampled three times each, whereas five turkey farms and one fish farm each were sampled twice. For each of these eight farms, the sample yielding the highest number of sequenced read pairs was chosen to represent the farm.

In order to remove low-quality nucleotides as well as adaptor sequences, DNA sequence read pairs were quality- and adapter-trimmed with the BBMap (v36.49) tool BBduk2 with the following arguments: qin=auto k=19 mink=11 qtrim=r trimq=20 minlength=50 (21).

Samples originating from 69 samples were sequenced more than once, usually due to low read throughput during the initial sequencing. In these cases, trimmed read pairs from all sequencing runs associated with the same sample were concatenated into combined files representing the individual farms.

Trimmed reads from each sample were aligned to the 3,081 ARGs in the ResFinder database (Bitbucket commit d3d7a6c) (22) and, separately, to a merged database of genomic sequences, as outlined below using the *k*-mer alignment software KMA (17) (v1.2.8). KMA was specifically designed to perform well when mapping against redundant databases such as the ones we used in this study, which contain high levels of sequence redundancy.

ResFinder offers a manually curated database of acquired ARGs, i.e., non-intrinsic genes discovered to provide bacterial AMR, with each ARG belonging to a specific AMR group and class (Data S3). Sequence reads were aligned to the ResFinder database using the KMA parameters “-mem_mode -ef −1t1 –cge -nf -nc.”

The database of genomic sequences was created by merging the following genome collections from NCBI and other sources into an indexed KMA database as previously described (23). Briefly, the latest versions of plasmids and bacterial, archaeal, human, fungal, parasite, and protozoan genomes were downloaded from NCBI (24) and supplemented with the genome collections from MetaHitAssembly (25), the Human Microbiome project (26), KVIT (27), and IMG-VR (28) as well as a previously described curated parasite database (29). This genome collection was indexed with KMA, and the trimmed reads were subsequently aligned using KMA parameters “-mem_mode -ef −1t1 -apm f -nf -nc.”

### Abundance data transformations

Both bacterial taxonomy and ResFinder matrices (Data S4 to S6) were subjected to zero replacement using the “SQ” method as implemented in the cmultRpl function in the zCompositions R package (version 1.3.4) (30).

The CLRs were calculated based on those and used for, e.g., principal component analysis (PCA) and differential abundance analysis with ALDEx2. Each sample ALR for ResFinder abundances was calculated as the log ratio between the length-corrected gene abundances (nominator) and the number of bacterial fragments (denominator). Such ratios were calculated at both the level of drug classes and the individual gene clusters.

### Quality control of ARG-assigned reads

To filter out low-coverage alignments before the downstream analysis of ARGs, we required that the ResFinder reference genes were at least 20% covered. Alignments with lower proportions covered had their mapped fragment count for such references set to zero.

### Quality of samples from fish farms

For many of the data sets originating from fish farms, only a low number of sequence fragments could be assigned to bacterial genomes or the ResFinder database of ARGs.

We opted, however, to not completely exclude them from all analyses. Fish samples were included in PCA ordination, hierarchical clustering, and core resistome analysis if they scored higher than the least informative (i.e., lowest-scoring) sample from pig and broiler herds in at least one of the three measurement criteria: (i) absolute number of fragments mapping to the ResFinder database, (ii) proportion of all fragments mapping to the ResFinder database, and (iii) estimated Chao1 richness of ARGs.

### Data analysis and visualization

All analyses of read mapping results were conducted using the open-source statistical environment R (31) version 4.0.1 (2020-06-06). Most figures were created with the R package ggplot2 (v2.3.3.2) (32). Exceptions are the heatmap in Fig. S3, which was created using the R package pheatmap (v1.0.12) (33), the network layouts in Fig. 5 created with gephi (v.0.9.2) (34), and the universality result panels in Fig. 6 made with R (v3.6).

### ARG alpha diversity and richness estimation

We estimated the richness (number of different ARGs) in each sample using the bias-corrected form of the Chao1 index: $S_{Chao1}=\;S_{obs}+\;\frac{f_{1}\left(f_{1}-1\right)}{2\left(f_{2}+1\right)}$, where $S_{obs}$ is the total number of different ARGs observed in the sample, $f_{1}$ is the number of ARGs with exactly one mapping sequence fragment, and $f_{2}$ is the number of ARGs with exactly two mapping fragments. The fraction $\frac{f_{1}\left(f_{1}-1\right)}{2\left(f_{2}+1\right)}$ is added to the observed richness $S_{obs}$ in order to account for low-abundance ARGs that were not detected during sequencing and mapping (35).

Alpha diversity was calculated as Shannon’s diversity index on the proportion of features with non-zero fragments sum-scaled to 1 (*p*): $H^{\prime }=-\sum p_{i}\ln p_{i}$.

### Core resistomes

We established core resistomes for each category, i.e., sets of ARGs that appear in the large majority of herds sampled for each host species (90% of sampled herds). For broilers and pigs, core ARGs are genes that appeared in at least 162 sampled herds (out of 178 broiler herds or 181 pig herds). Because fewer turkey and veal calf herds were sampled, the minimum number required was only 54 herds (out of 60 turkey flocks or 61 veal calf herds). Only a few fish data sets passed the quality threshold; therefore, core fish ARGs needed to appear in at least 13 out of 14 sampled fish herds.

Core resistomes were also determined for each country cohort within all host species except fish. Here, core ARGs are genes that were detected in at least 18 herds of the same host species and from the same country.

### Co-occurrence analysis of bacterial taxa and ARGs

To enable co-occurrence visualization in a network interface, we constructed a correlation matrix by calculating all pairwise Spearman’s rank correlations between bacterial genera and ARGs as well as between ARGs themselves. Zero correlations and bacteria–bacteria correlations were excluded to focus on bacteria–ARG and ARG–ARG correlations. Significant Benjamini–Hochberg correlations, corrected for multiple testiing (*P* < 0.01 and |ρ*| >* 0.8) were retained for constructing an undirected network where node sizes represent the calculated betweenness centrality and nodes were laid out using the Fruchterman–Reingold algorithm using gephi (v0.9.2) (34).

### Questionnaire data

General farm characteristics, antimicrobial use (AMU group treatments), and information about biosecurity were retrieved from a standardized questionnaire completed by the farmer as published previously (14, 15, 36). The usage of antimicrobials on farms of pigs, broilers, turkeys, and veal calves has been elaborately described elsewhere (37–39).

Aggregated biosecurity scores per farm were retrieved from previous studies (for pigs [14] and broilers [15]) or newly calculated from the questionnaire based on the algorithm of the Biocheck.UGent scoring system (for veal calves [40]).

### Log ratio transformation and PCA

Respecting the compositional nature of quantified ARGs (18), we transformed the filtered ARG count matrices from simplex space into real space using a log ratio transformation. In that way, true Euclidean distances between resistome compositions can be calculated as required by PCA. Because we are dealing with complex samples from different environments, we chose the IQLR transformation in order to try and correct for the asymmetric presence and absence of ARGs in the different host species cohorts (41). We used the R package ALDEx2 (v2.1.20.0) (42) to identify a set of ARGs with the medium variance within each of the four non-fish host species (i.e., variance between the first and third quartiles of variance). The geometric mean abundance of these ARGs was used as a denominator in subsequent log ratio transformations.

During such transformations, ALDEx2 estimates the technical variation of gene abundances by drawing 128 Monte Carlo samples from a Dirichlet distribution whose parameters are based on the provided fragment count matrix. While this inferred technical variation is crucial for robust differential abundance testing, it cannot be reasonably visualized in the ordination plots, heatmap, or box plots we created. Therefore, we calculated the mean of the 128 resulting log ratio matrices for use in visualizations of log ratio values.

These mean log ratio values were used to visualize the samples’ beta diversity: (i) in an ordination plot using PCA with Euclidean distances and (ii) in a heatmap displaying Ward linkage clustering of Euclidean distances.

The mean ALR ARG abundances were visualized per country and species in box plots. Overall, per species, country resistome differences were compared by performing a classic or Welch’s analysis of variance (ANOVA) (43, 44). In case of a significant difference, *post hoc* tests were carried out (i.e., respectively, Tukey’s honest significant difference test [Tukey HSD] [45] or a Games–Howell *post hoc* test [46]).

### Risk factor random-effects meta-analyses in pigs, broilers, and veal calves

For pigs, broilers, and veal calves, respectively, 176, 176, and 59 farm data sets with matching questionnaires (e.g., AMU and biosecurity) and ARG data were available for analysis (14, 15, 36). We used R v3.6.3 for all risk factor analyses (47). A random-effects meta-analysis by country was run for veal calves using the R package metafor (48) (v2.4.0) as described and calculated previously in pigs and broilers (14, 15).

### Dispersal limitations of livestock ARGs

If ARGs that co-occur in different farms tend to exist in similar ratios to each other, it suggests that they are similarly selected across the farms (49). Such a pattern, therefore, implies that interfarm variability is created via colonizing ARGs or dispersal limitations. Overlap, a measure for ARGs shared between samples, and dissimilarity, as a measure of variation in abundances of the shared ARGs, were calculated for all sample resistome combinations and grouped in combinations of inter- and intra-livestock species and country comparisons. Subsets of samples can vary in the degree that pairings sharing ARGs predict the relative abundances of these ARGs or the strength of the universality signature; this is named “group dynamics.” If each of these subsets of samples is still universal, the data set as a whole is universal.

Each pair of sample resistomes was compared in order to determine the relationship between their overlap and dissimilarity. Overlap was calculated as $\left(\tilde{x},\tilde{y}\right)=\;\sum_{i\in S}\frac{{\tilde{x}}_{i}+\;{\tilde{y}}_{i}}{2}$, where ${\tilde{x}}_{i}$ and ${\tilde{y}}_{i}$ are detection (1) and non-detection (0) of ARG *i* in sample *x* and *y,* respectively. An overlap coefficient of 1, thus, indicates complete concordance between the observed ARGs in a pair of samples (*S*), like previously shown for bacterial taxa (49).

We used a dissimilarity index that disregards overlap and only considers the quantitative dissimilarity; the root Jenson–Shannon divergence $(\tilde{x},\tilde{y})=\sqrt{\frac{D_{KL}\left(\tilde{x},\frac{\hat{x}+\hat{y}}{2}\right)+D_{KL}\left(\tilde{y},\frac{\hat{x}+\hat{y}}{2}\right)}{2}}$, where the DKL is the Kullback–Leibler divergence, calculated by $D_{KL}\left(P,Q\right)=\sum_{x\in X}P\left(x\right)ln\left(\frac{P\left(x\right)}{Q\left(x\right)}\right)$ (44). A multi-membership model was fitted using Monte Carlo Markov Chain (MCMC) methods to these pairwise metrics of overlap and dissimilarity (50). This type of Bayesian model accounts for each pairing not being completely independent and can also determine whether covariates like country and host species differ between sample pairs. The R package MCMCglmm was used with an uninformed inverse-Wishart prior with 10,000 iterations (51).

The three model equations used for the multi-membership models were


 Model A: log⁡( dissimilarity )∼log⁡(1− overlap )

 Model B: log⁡( dissimilarity )∼log⁡(1− overlap )∗ Same country +log⁡(1− overlap )∗ Same species 

 Model C: log⁡( dissimilarity )∼log⁡(1− overlap )∗ Species Country Category 

Full mapping and alignment results in the compact KMA output “mapstat” format as well as full taxonomic annotation for the database of genomic sequences are available at Zenodo (https://doi.org/10.5281/zenodo.5880380). Sample metadata is available in Data S2 and mapped count data to Resfinder; the bacterial database and other databases can be found in Data S4 to S6.

Helper R functions to work with the mapstat files can be found on GitHub (https://github.com/genomicepidemiology/mapstatHelpers). The analysis code used to calculate abundances, compare the resistomes statistically, and visualize them can be found here (https://github.com/genomicepidemiology/livestock-amr-analysis).

## RESULTS

A total of 23.44 billion PE reads were obtained from the 548 samples (average 42.77 million PE reads, range 0.62–183.71 million). There were large differences between species, with rainbow trout having the lowest sequencing depth with a mean of 21.42 million PE reads (Data S6).

### The acquired livestock resistome

**Fig 1:**
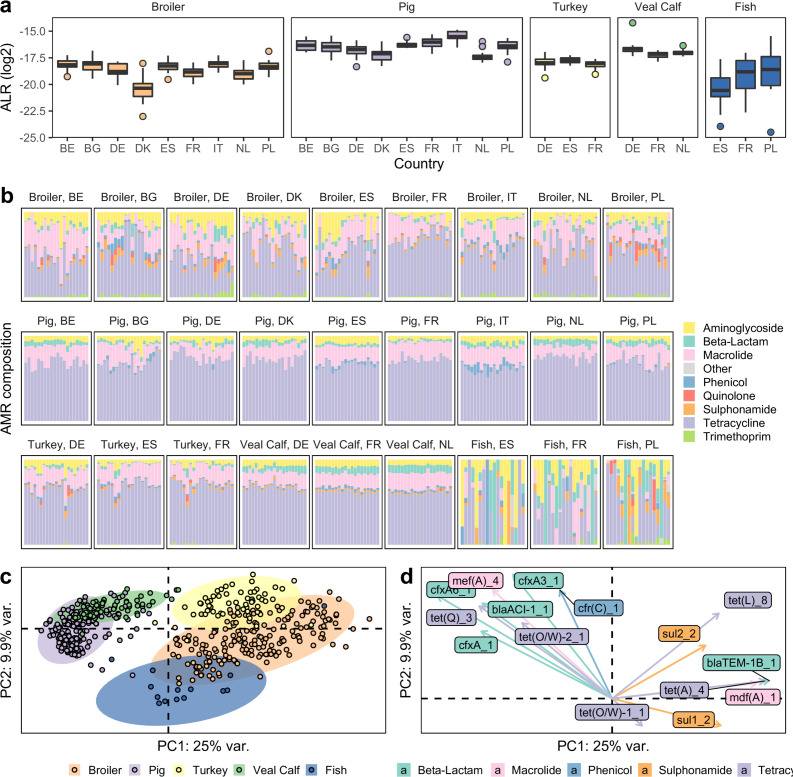
AMR in different livestock cohorts. (a) Summed abundance of AMR genes across the livestock species (log2). Horizontal box lines represent the first quartile, the median, and the third quartile. Whiskers extend to the smallest and largest data points within the interval (first quartile − 1.5 × interquartile range [IQR], third quartile + 1.5 × IQR). Data points outside this interval are plotted as circles. (b) Class-level AMR composition. Samples are grouped into panels by host species and country; each sample is represented by a stacked vertical bar. Colors indicate the relative number of gene counts corresponding to each AMR class. (c and d) AMR gene-level biplot, based on mean IQLR values obtained with ALDEx2 and Euclidean distances in the resulting simplex space. Low-quality fish samples are excluded (44 excluded out of 58 total). (c) Projection of livestock samples onto the first two principal components. Ellipses show the 95% confidence interval for each host species (multivariate *t*-distribution). (d) Corresponding projection of AMR genes onto the (same) first two principal components. The 15 genes with the highest variance in IQLR space are shown and colored according to their AMR class.

From 6.71 * 10^−6^ to 0.55 % of the reads per sample mapped to ResFinder (Fig. S1). On average, the highest AMR loads were observed in pigs, followed by veal calves, broilers, turkey, and fish (Fig. 1a). The total AMR load varied between farms and countries for all animal species, of which the country effect was mostly visible in broilers (Fig. 1a). For pigs, only the mean AMR abundance in Italy was significantly higher than in the Netherlands (*P* < 0.05, ANOVA and Tukey HSD). For broilers, the mean AMR load varied significantly among the countries, e.g., the mean AMR abundance in Belgian farms was found to be significantly higher than in Denmark, France, and the Netherlands (*P* < 0.01, Welch’s ANOVA and Games–Howell test). There was no significant difference in mean AMR abundances between the subset of countries with turkey, veal calves, and trout data sets (*P* > 0.05, one-way ANOVA or Welch’s ANOVA).

The relative contribution of AMR stratified by sample and antimicrobial drug class can be seen in Fig. 1b. For fish, only a limited number of reads were assigned to the resistome, and a large variation was observed (Fig. S1 and S2). For the remaining livestock, the relative abundance of tetracycline ARGs was clearly most common across all animal categories, followed by macrolide and aminoglycoside ARGs. Sulfonamide ARGs were almost absent among pigs but consistently found among veal calves and also frequently among broilers and turkeys. Beta-lactam resistance genes were most common among veal calves, and in general, the class-level AMR compositions in calves were very homogenous.

A total of 534 different ARGs were observed across the samples. Using PCA on the gene-level resistome, we saw clear host species clustering, with 35% variance explained on principal components 1 and 2 (Fig. 1c). However, pig and veal calf resistomes clustered very closely together, as did the broiler and turkey resistomes, despite higher within-species dispersion in poultry.

The first principal component essentially split the resistomes according to the mammalian or avian origin, with pig and veal on the left and broiler and turkey on the right. The second component was better at separating the newly added turkey and veal calf resistomes, from the pig and broiler resistomes that had centroids lower on this axis. The fish resistomes were best separated from the other populations along the second component and were overlapping with other animals along the first component.

We determined the individual ARGs contributing most to this separation (Fig. 1d). The mammal hosts were strongly associated with multiple ARGs, like *mef*(A), *tet*(Q), *bla*_ACI_, and different *cfx*A variants. Sulfonamide resistance genes *sul*1 and *sul*2 were associated with broiler and turkey, respectively, and helped drive their separation along the second component. *tet*(A) and *mdf*(A) positively associated with the first component. A similar pattern was observed in a heatmap (Fig. S3).

### The conserved core of livestock resistomes

**Fig 2:**
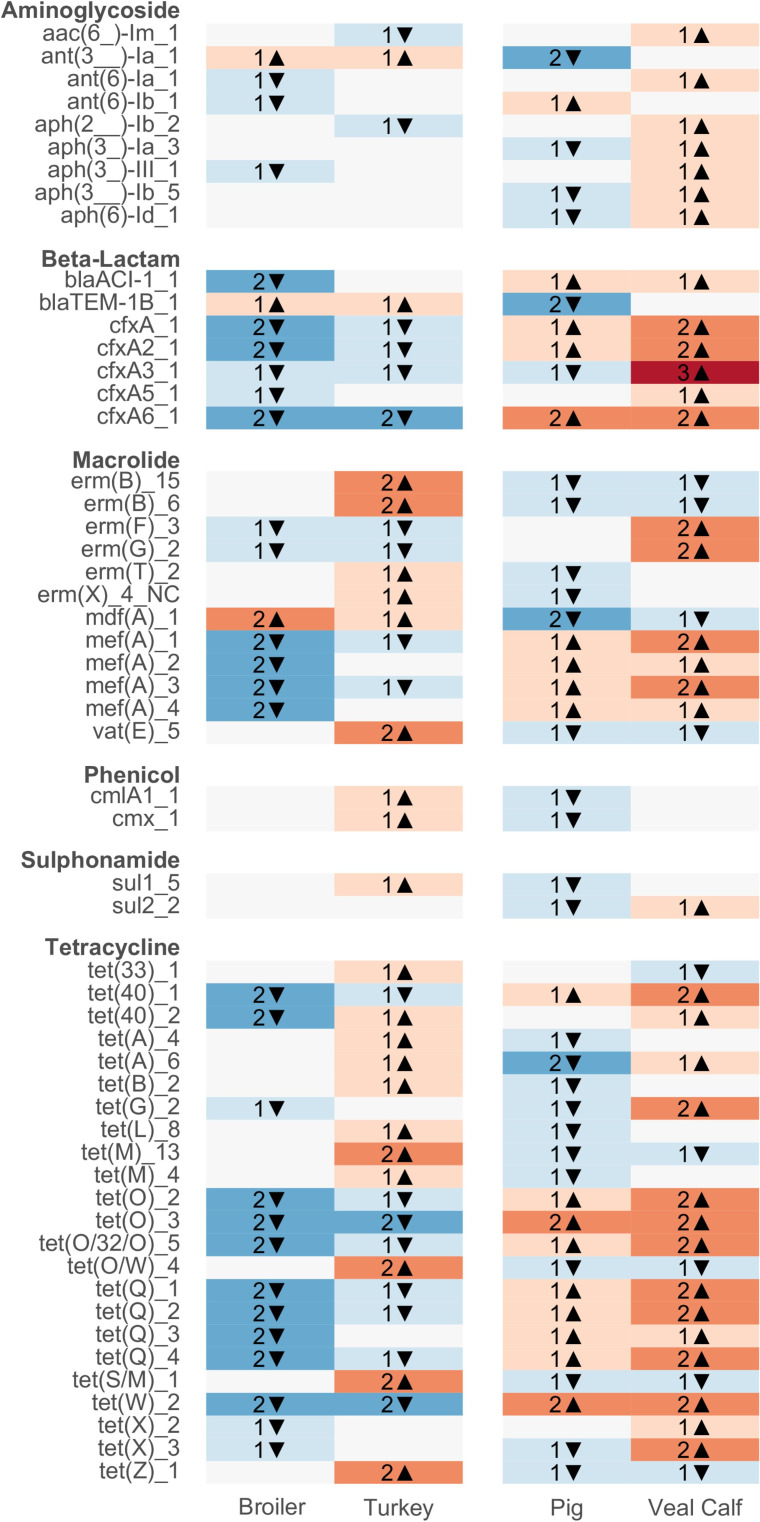
Animal host effect on AMR genes. Color shades represent relatively high (red) and low (blue) gene abundances in herds of the corresponding host species. Text labels indicate the number and direction of pairwise differential abundance tests flagged as “substantial” (ALDEx2: absolute effect size >1 and overlap <0.05) for this gene and host species. Only AMR genes with at least one substantial pairwise result are shown.

We determined the ARGs that were core to each host animal and animal–country combination. Certain ARGs were so widespread that they were core to every country and animal species and include variants of *ant(6)-Ia*, *aph(3)-Ia*, *erm*(G), and *tet*(W) (Fig. 2; Fig. S4 and S5). Turkeys had more core ARGs than veal calves, which had more than the remaining animals (Fig. S4). Quinolone ARGs were core to Spanish and German, but not French turkey flocks. The latter had many more trimethoprim resistance core genes, not unlike broiler resistomes. Interestingly, the Netherlands, which had both low AMR levels and relatively few core ARGs in broilers and pigs, actually had the most core genes in veal calves specifically (Fig. S4).

Broilers showed a large threefold difference in the number of animal–country core ARGs from ~30 in Denmark to >90 in Italy. The more limited sampling effort in veal calves and turkeys of course also limits the likelihood of observing this, but it remains evident that broiler resistomes are very atypically diverse across Europe compared to other livestock species, which one might suspect could be due to a large degree of environmental contamination for those samples. But even if floor sampling risks contaminating the feces slightly, floor sampling has been shown to be highly comparable to fecal sampling, and core analysis in general should be very resistant to random contamination (2).

Broilers had relatively few core ARGs, even compared to turkey, which are more similar in terms of beta-diversity. Surprisingly, veal calf, pig, and turkey resistomes shared quite a few core genes that were not core to broiler.

**Fig 3:**
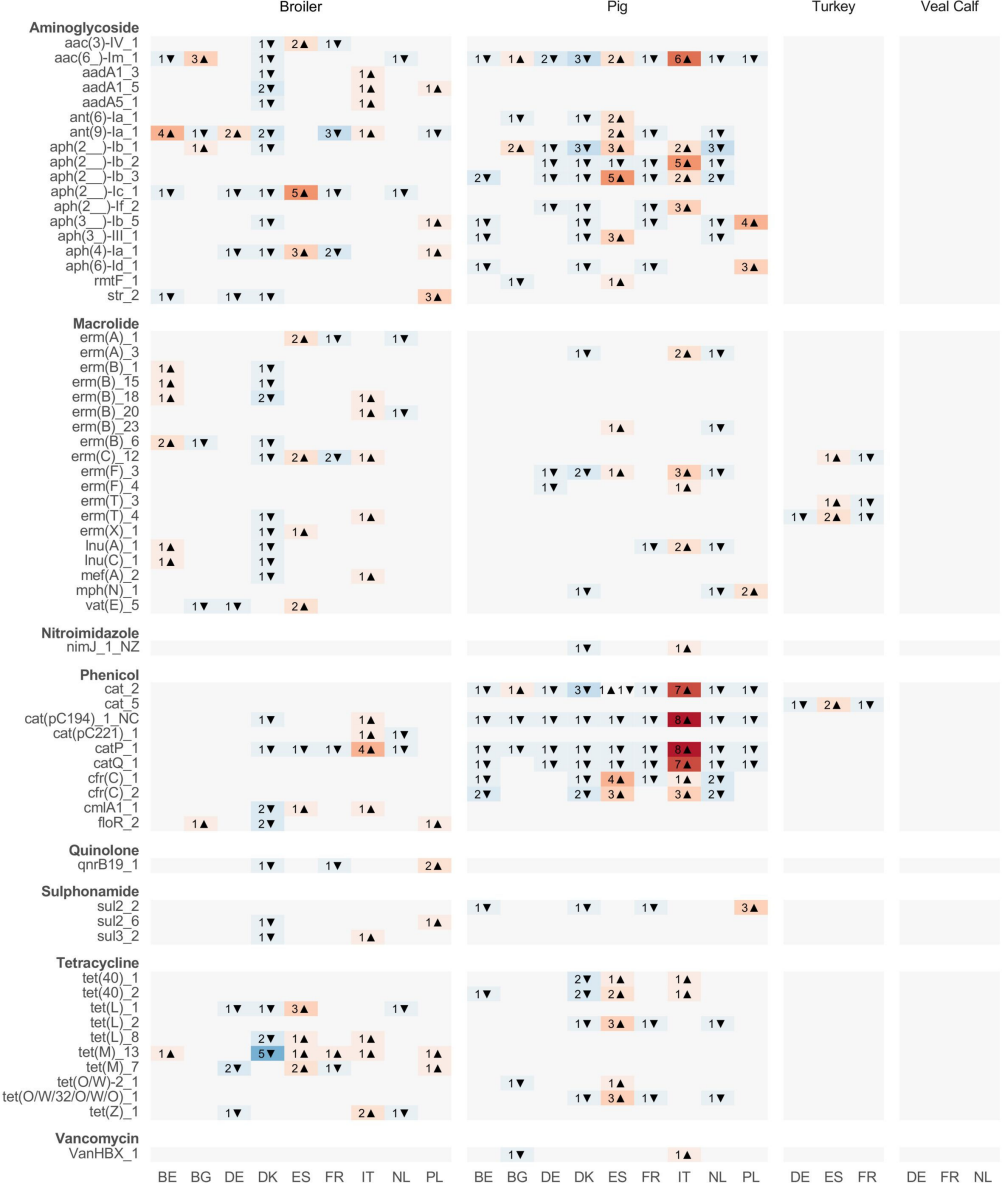
Country effect on AMR genes within different host species. Color shades represent relatively high (red) and low (blue) gene abundances in herds from the corresponding country. Text labels indicate the number and direction of pairwise differential abundance tests flagged as “substantial” (ALDEx2: absolute effect size >1 and overlap <0.05) for this gene and country. Only AMR genes with at least one substantial pairwise result are shown (no such result was detected in veal calf herds).

Several ARGs were very characteristic for a single animal category in a single country (Fig. 3; Fig. S6). The *erm*(43) ARG was core to only Danish broilers. *aph(6)−1d* was core only to French veal calves, while *erm*(33) and *erm*(22) were core only in Spanish turkeys.

### Differential abundance analysis

Core ARGs can mark strong differences when certain ARGs are completely absent in certain environments but cannot reveal more subtle differences in quantitative resistome composition. We, therefore, performed differential abundance analyses to identify ARGs statistically over- and under-represented in species or species–country resistomes.

Many ARGs differed significantly in relative abundance between livestock species and specifically in relation to the recently added turkey and veal calf samples (Fig. 2, Fig. S7 and S8). Noticeably, *tet*(M), *erm*(B), and *vat*(E) were overrepresented in turkey samples and *cfx*, *mef*(A), and *tet*(Q) in veal calves.

We also tested for country effects on mean ARG abundance in each animal cohort (Fig. 3). For the turkey, *erm*(C), *erm*(T), and *cat* ARGs were more abundant in the samples from Spain than samples from France and sometimes Germany (Fig. S8). No ARGs were individually overrepresented in turkey resistomes in the two latter countries.

In broilers and pigs, the number of differences is too numerous to list here (Fig. S9 and S10). *qnr*B19 was highest in Polish samples and statistically higher than Danish and French samples. *floR* was higher in Poland and Bulgaria than Denmark. The *tet*(M)_13 variant was significantly lower in Denmark than in five of the eight other countries. Lastly, and as previously shown, multiple chloramphenicol ARGs were overrepresented in Italian samples, including *cat*, *cat*P, and *cat*(pC194).

### Alpha diversity and richness

**Fig 4:**
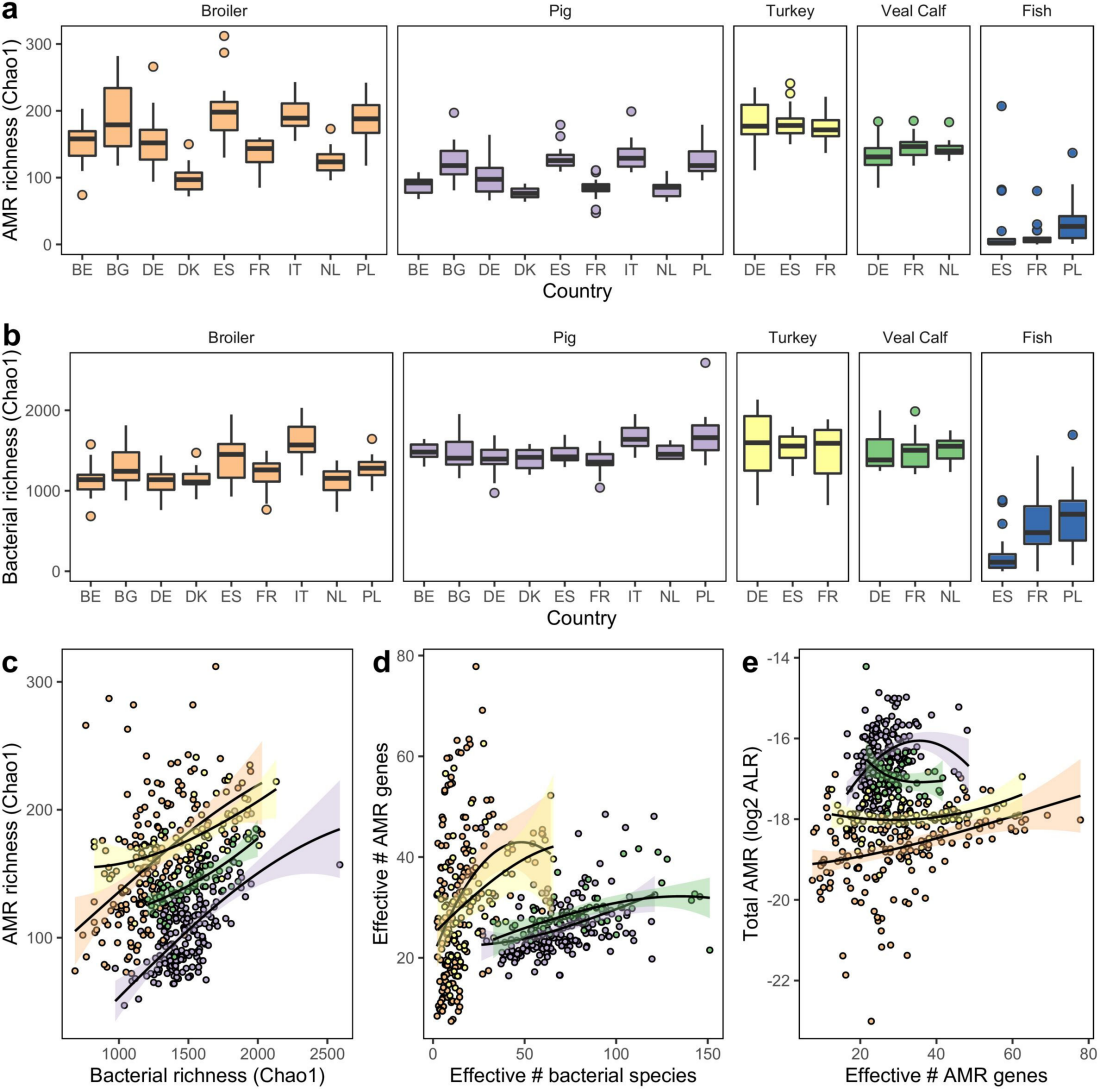
Alpha diversity of livestock microbiomes. (a) Estimated richness (Chao1) of AMR genes, separated by host species and country of origin. (b) Estimated species richness (Chao1) of bacterial species, separated by host species and country of origin. (c) Association between bacterial and AMR estimated richness from previous plots. (d) Association between bacterial species and AMR gene diversity, calculated as effective number of species exp(Shannon). (e) Association between AMR diversity and total AMR load. (c–e) Local regression lines (loess) are drawn separately for each animal species and mark extrema on the *x*-axis. See panel a for the meaning of the color.

We calculated the expected richness (Chao1) for both ARGs (Fig. 4a) and bacterial species (Fig. 4b) in the samples. Generally, there was a large overlap across countries between the range of diversities for the newly added turkey and veal calf samples. For the avian samples, ARG richness was more variable both within and between countries, compared to any of the other livestock species. The fish microbiomes scored very poorly in terms of richness, both for ARGs and bacterial species.

At comparable bacterial richness levels, we saw the highest ARG richness in broilers and turkeys. While only spanning a limited range, at similar bacterial richness levels, veal calves had ARG richness somewhere between the rich poultry resistomes and the poorer pig resistomes (Fig. 4c).

In terms of actual species and ARG diversity (effective number of species), the mammalian species looked much more comparable (Fig. 4b and c; Fig. S11). Even the most diverse bacteriomes of the avian species were only comparable to low-diversity mammalian samples. The relationship between bacterial and ARG diversity was positive for all four livestock species.

Generally, low bacterial species richness was associated with low ARG richness (Spearman’s rho: 0.258, *P* = 5.91 × 10^−9^). Importantly, the associations for the different host species had different intercepts, showing that the association is not simply a product of bacterial diversity and there might be species-specific optimums one could identify.

### Co-occurrence patterns of bacterial genera and ARGs

The co-occurrence patterns among ARGs and between bacterial genera and ARGs were explored using network inference based on strong (>0.8) and significant (Benjamini–Hochberg adjusted *P*-value <0.01) correlations. We first analyzed a combined network graph with all animals, but the fish. Many mainly positive correlations were observed between bacterial genera and ARGs or in between ARGs Several indications for multi-drug resistance were found among which *Escherichia* linked to beta-lactams (*bla*_TEM_), aminoglycosides (*aad*, *aph*), and macrolide (*mdf*) ARGs in broilers and turkeys. Other indications for multi-drug resistance are ARG modules indicative for frequently co-occurring ARGs, best observed, for instance, between mostly the *tet(33, 40, A, O, Q), bla*_TEM_*, dfa, mdf, aad, cmx,* and *sul* genes. Both *bla*_TEM_ beta-lactam and *mdf*(A) macrolide ARGs are frequently enriched at the cost of several mainly Gram-positive anaerobic taxa (or vice versa).

**Fig 5:**
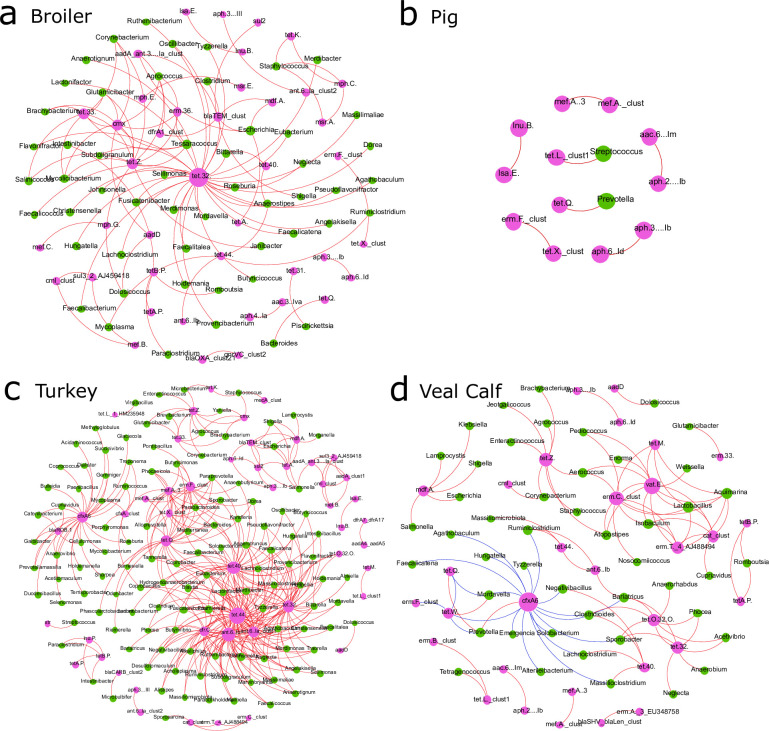
Co-occurrence correlation networks of bacteria and ARGs within terrestrial livestock. Fruchterman Reingold network representation of Spearman’s rank correlations (rho > 0.8, and Benjamini–Hochberg-corrected *P* < 0.01) between bacterial (genera) and ARG (ARGs clustered on 90% sequence identity) as well as within ARG taxa. Nodes represent bacterial (green) and ARG (pink) taxa (node sizes reflect the degree of connectivity). Edges represent either positive (red) or negative (blue) Spearman’s correlations between taxa. (a) Co-occurrence networks for broilers (a), pigs (b), turkey (c) and veal calves (d) can be seen.

Stratifying by animal cohort, there was a strikingly large difference in the number of nodes for pigs, where only 2 bacterial and 12 ARG nodes are observed, contrasting the networks of other livestock harboring over 50 nodes (Fig. 5). Another striking feature is the occurrence of two inversely correlated modules of ARGs and bacteria. In veal calves, the mutual exclusiveness of the beta-lactamase ARG *cfx*A6 and several bacterial genera is quite prominent.

### Evidence of dispersal and universality of ARGs

**Fig 6:**
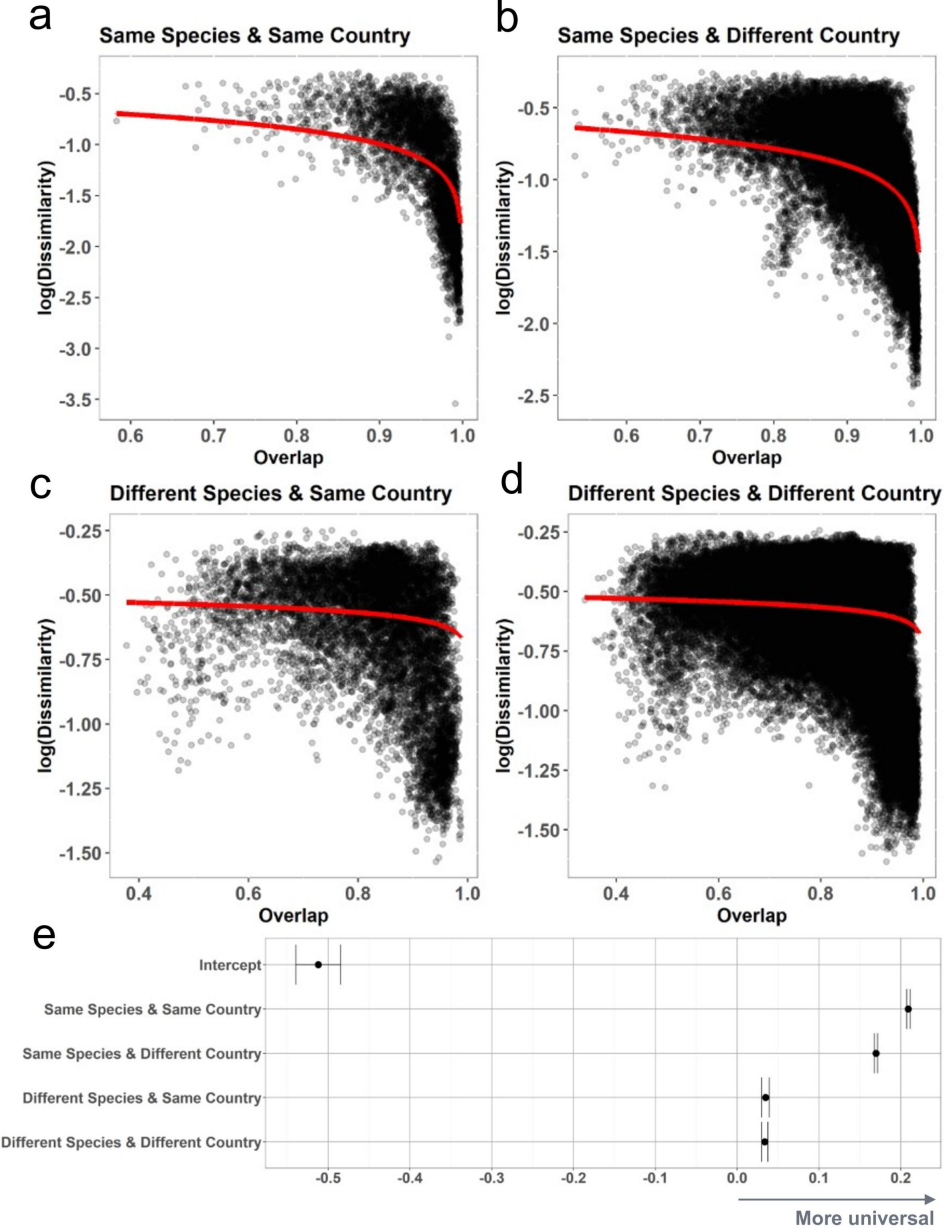
Universality dynamics of ARGs in terrestrial livestock. (**a–d**) Scatter plots and local trend lines for associations between resistome overlap (*x*) and dissimilarity (***y***) in farm sample pairs, stratified by same (**a, c**) and differing (**b, d**) countries (columns) and same (**a, b**) and differing (**c, d**) livestock species status (rows). (**e**) Model coefficients for $\log\left(dissimilarity\right)\sim \log\left(1-overlap\right)*Category$ where category encodes whether covariates are shared. On the *x*-axis, shifting further to the right suggests a more universal or stronger effect on the negativity of the dissimilarity–overlap curve.

As seen in Fig. 6, the livestock resistomes are universal (influenced by dispersal limitations), with some groupings within the data having a stronger signature of universality than others (group dynamics). This evidence of universality is illustrated by a negative sloping model curve of dissimilarity and overlap shown in all categories. Pairs of samples from the same species are more universal than those from different species, with samples from the same species and the same country appearing most universal. These results highlight the species barrier to the dispersal of ARGs, with ARG dispersal happening more freely in the same species across countries than between different animal categories in the same country.

### Risk factor analyses in pigs, poultry, and veal calves

The original quantification of AMR loads, stratified by drug class, in the pig and poultry herds has previously been used for risk factor analyses (14, 15). Now that we have updated the quantification pipeline, databases, and gene filtering and are treating the data compositionally correct to minimize the risk of spurious associations, it was possible that associations with previously published and identified risk factors would change in magnitude and precision.

Modeling the updated AMR class-level abundances reported here with the risk factors resulted in similar effect sizes as published before, but a slight decrease of all estimates (β) was found.

For the pig herds, all previously significant AMU–AMR associations (14) were confirmed (false discovery rate [FDR] adj. *P* < 0.05) in the analyses using the updated data sets, with the exceptions of a positive associations between lincosamide usage and macrolide AMR and tetracycline usage on corresponding tetracycline ARGs (Data S8, ALR data).

For poultry, all previously significant associations between AMU and AMR (15) could be reproduced except for the significant associations with internal biosecurity (Data S8, ALR data). Estimates of prior analyses only slightly differed from previous estimates.

In veal calves, a negative association was observed between colistin use and acquired resistance genes. In addition, at farms in which no cleaning of housing was performed (compared to farms that used soaking agents and disinfectants), the calves carried lower phenicol resistance levels in their feces (FDR-adjusted *P* < 0.05).

## DISCUSSION

Recently, a number of studies have reported on the resistome of different animal species, mainly pigs, broilers, and cattle (12, 52–56). However, to our knowledge, this study is the first to conduct a comparative analysis of the resistome of all major food-producing animal sectors from the same countries.

We obtained a low abundance of bacterial reads from fish. This might be due to how fish defecate and the clearance period prior to sampling. Thus, too little feces material was frequently present, making fish fecal microbes undersampled in the data set compared to other sources. This and other complications like high water contents make microbiome studies of fish feces difficult, and other proxies like skin scratches should be considered (57).

We found the highest abundance of AMR in pigs and veal calves, followed by broilers, turkey, and fish. Resistome beta-diversity largely mimicked the phylogenetic relationship between host animals, with intra-group similarity of both mammal and avian species.

Importantly, we also observed that different variants of the same ARG were frequently found in different species and contributed significantly to the separation of the resistomes for veal calves/pigs versus broilers/turkey. This suggests that future studies investigating the evolution and epidemiology of the resistomes should consider not only each specific ARG or AMR drug class but also the full variation underlying the resistome. Co-occurring ARGs were in line with previous findings, with several identified core resistome genes [*ant*(6)*, aph(3), tet*(W), and *erm*(B)] and earlier observations in environment/wastewater from livestock farms (58) among which the ARG clusters *tet*(33)*, cmx, aadA,* and *sul* are dominant in the overall network as well as in turkeys and broilers.

Recent studies based on human clinical isolates, urban sewage, and experimental and modeling approaches have suggested that the abundance of ARGs is not only a consequence of AMU but also associated with socio-economic factors most likely influencing increased transmission (59–63). Considering whether the effect of the spread of new ARGs (dispersal) is important, or if dispersal effects are minimal compared to large variability in local selection pressures across countries, is an important question in managing future resources for tackling AMR. Our universality analyses suggested that dispersal limitations do impact the abundances of livestock AMR and also that these dispersal limitations are strongest between animal categories. This suggests that limiting transmission between farms, animal hosts, and countries should also be an important factor in controlling AMR in the future. It should, however, be noted that our analyses are based on pooled samples from a limited number of farms per country.

Though most significant effect sizes in risk factor analyses agreed with the conventional wisdom of directionality, there were also some that warrant further exploration. The observation that cleaning and disinfecting between batches of veal calves were associated with higher phenicol resistance is potentially important. Phenicol resistance is indeed often encoded by efflux pumps that target a wide spectrum of both classical antimicrobials and disinfectants, making them concerning drivers of cross-resistance. Thus, there is a potential mechanistic explanation for our observation, and we think it would be prudent to further verify with more calves from a more diverse company pool. The effect might be a worthy tradeoff, since cleaning has other known and established effects on transmission and disease prevention.

A high bacterial diversity has in multiple studies been shown to be associated with a healthy gut microbiome (64). We observed in our study that high bacterial diversity whether measured as estimated richness (Chao1) or effective number of species (exponent of Shannon) was associated with both higher abundance and diversity of ARGs. We would normally assume that antibiotic treatment would reduce gut microbiome diversity and allow for more room for the growth of resistant bacteria. Once the treatment is stopped, the microbiota will return to a new dynamic balance between the different bacterial groups. In humans, it has been shown that a large proportion of potential ARGs are not transmissible to pathogenic species (65), which could be one factor influencing our observation. Another possible explanation is that high bacterial diversity maximizes the risk that a specific present ARG is ready to be selected for as soon as a corresponding antimicrobial agent is used. This would fit observations regarding pest invasion of forests in North America showing a linear association between tree diversity and pest diversity (66). However, that study also suggested that as diversity further increases, then “pest” or ARG diversity and abundance would again decrease. It is also important to note that these associations were only clearly visible when stratifying data by livestock host species. It would be interesting to determine, in future work, the nature of the interaction between bacterial diversity, the diversity and abundance of ARGs, and health.

### Conclusions

We identified clear core resistomes that were highly significant for the specific animal species. Using updated reference databases, optimized metagenomic alignment methods, hit filtering, and treating each resistome compositionally correctly, previously reported significant associations between AMU, biosecurity, and AMR in pig and poultry farms in nine European countries were validated, suggesting that the trends are robust with respect to methodological changes and updated reference data. In veal calf farms in three countries, we found some evidence for associations between the use of antimicrobials and cleaning agents and increased AMR.

Our study suggests that while AMU is still an important driver for AMR, factors contributing to increased bacterial diversity and transmission between farms, countries, and animal species might also be associated with higher AMR load. Future efforts to fight AMR should consider bacterial diversity and continue to emphasize biosecurity measures.

## Data Availability

DNA sequences corresponding to the 359 metagenomic samples obtained from 181 pig herds and 178 broiler herds are available in the European Nucleotide Archive (ENA) via project accession PRJEB22062 (12). DNA sequences corresponding to the 60 turkey (16) and 61 veal calf samples are available via ENA project accession PRJEB39685. DNA sequences from the 58 rainbow trout samples are available from accession PRJEB42464.

## AFTER EPUB

*[This article was published on 19 March 2024 with a spelling error in the byline. The spelling of Alex Bossers was corrected in the current version, posted on 1 April 2024.]*

## SUPPLEMENTAL MATERIAL

Supplemental figures msystems.01328-23-s0001.pdf Figures S1 to S11.

Supplemental material msystems.01328-23-s0002.xlsx Additional metadata and detailed abundance tables.

ASM does not own the copyrights to Supplemental Material that may be linked to, or accessed through, an article. The authors have granted ASM a non-exclusive, world-wide license to publish the Supplemental Material files. Please contact the corresponding author directly for reuse.
