# Supplementary material for: The European livestock resistome
Source: mSystems. 2024 Mar 19;9(4):e01328-23. doi: 10.1128/msystems.01328-23 (PMC11019871; doi:10.1128/msystems.01328-23)
Supplement: Supplemental figures — Figures S1 to S11. [file msystems.01328-23-s0001.pdf]

Supplementary figures

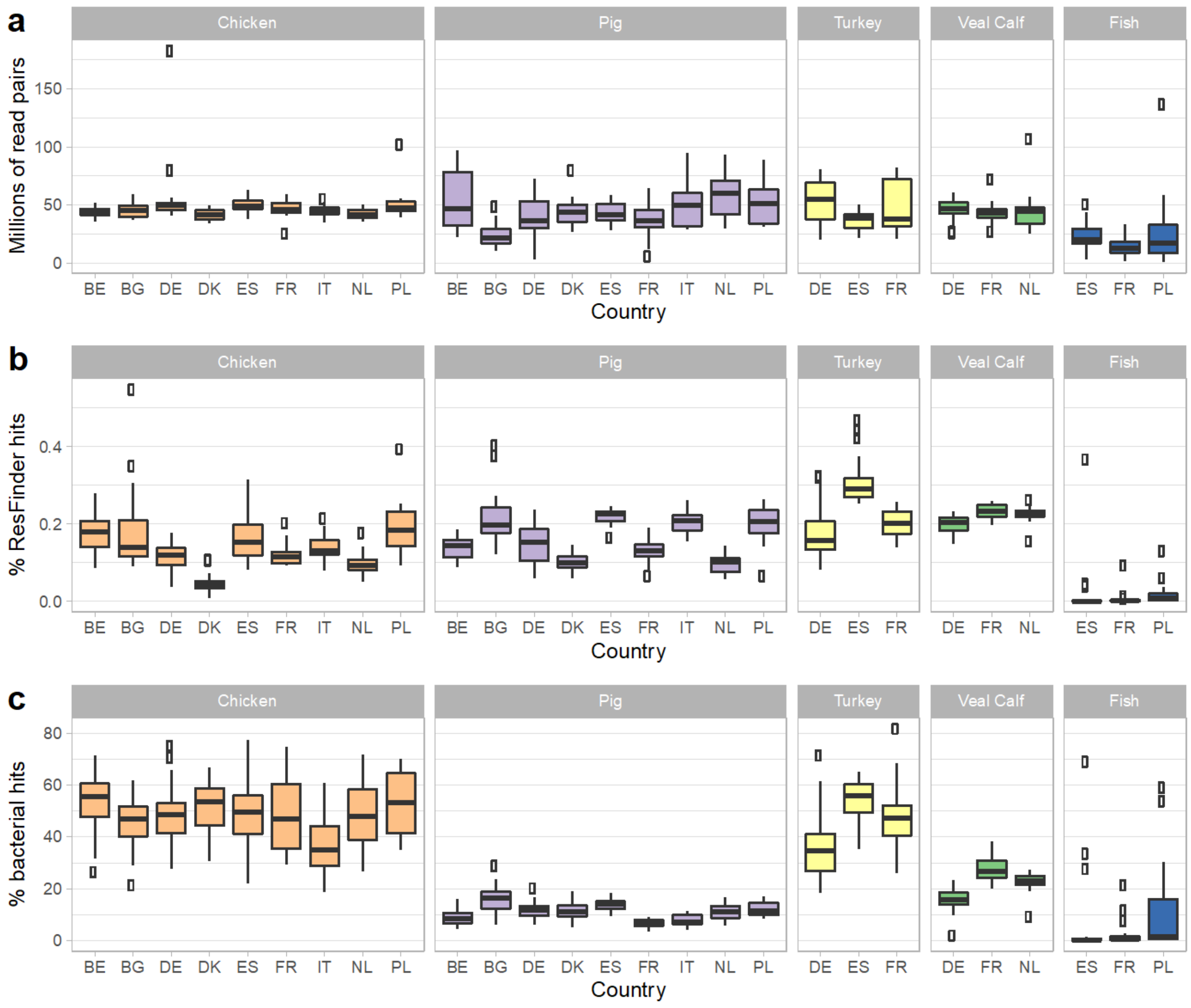

Supp. Fig. 1 | Sequencing throughput and mapping proportions. Horizontal box lines represent the first quartile, the median, and the third quartile. Whiskers extend to the smallest and largest data points within the interval [first quartile − 1.5 × interquartile range (IQR), third quartile + 1.5 × IQR]. Data points outside this interval are plotted as circles. **a:** Millions of read pairs per sample (after quality control and trimming). **b:** Percentage of input read pairs mapping to the ResFinder database. **c:** Percentage of input read pairs mapping to bacterial genomes.

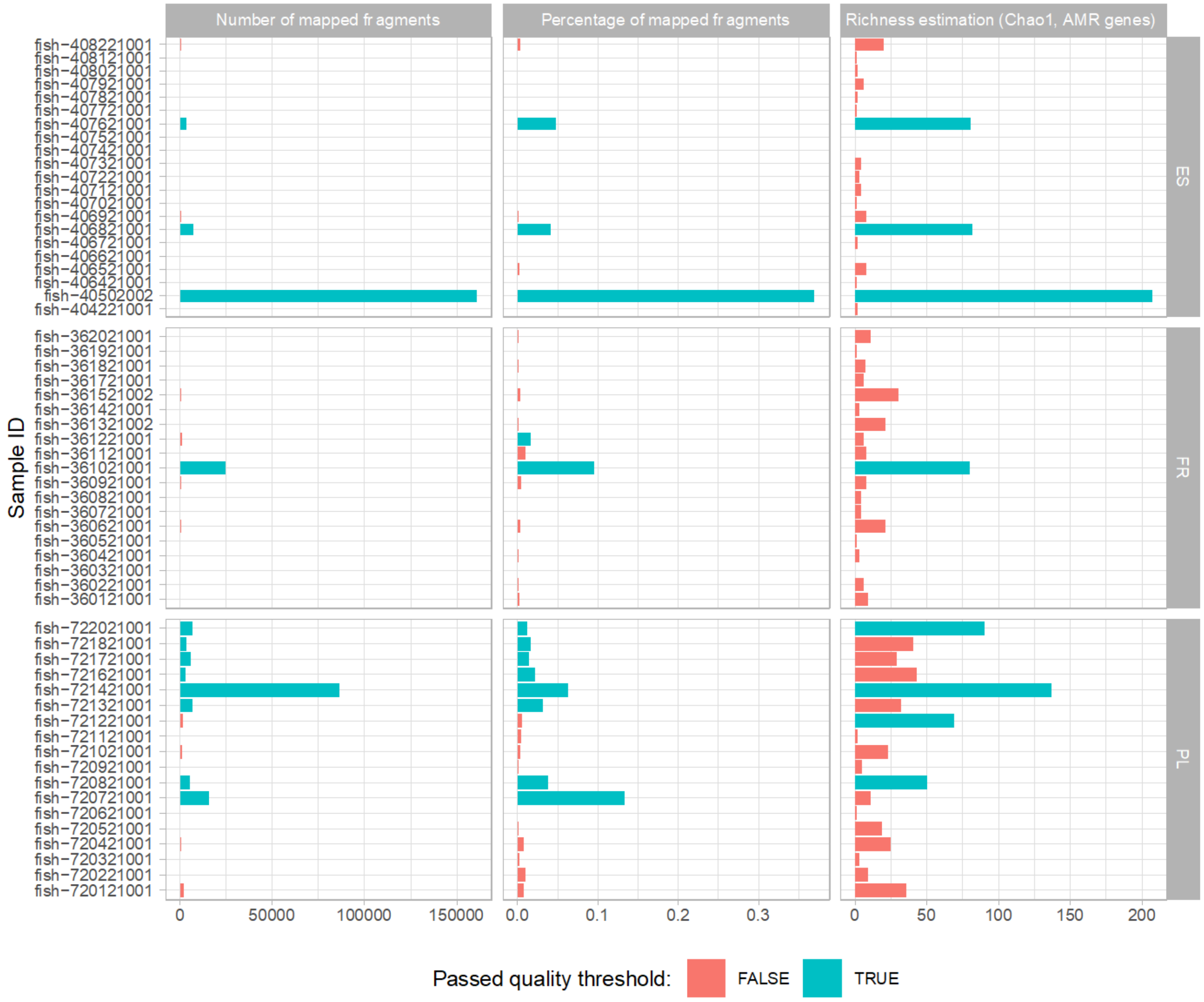

Supp. Fig. 2 | Quality assessment of fish samples. Absolute number as well as percentage of read fragments mapping to the ResFinder database are shown for each sample (left and middle column), along with the estimated richness of AMR genes in each sample (right column). Bars are colored depending on whether or not the sample passes the quality threshold for this particular parameter (2,318 mapped fragments; 0.00958 % mapped fragments; Chao1 index of 47). Fourteen of the 58 fish samples lie above the quality threshold of at least one parameter and therefore pass the quality filtering step.

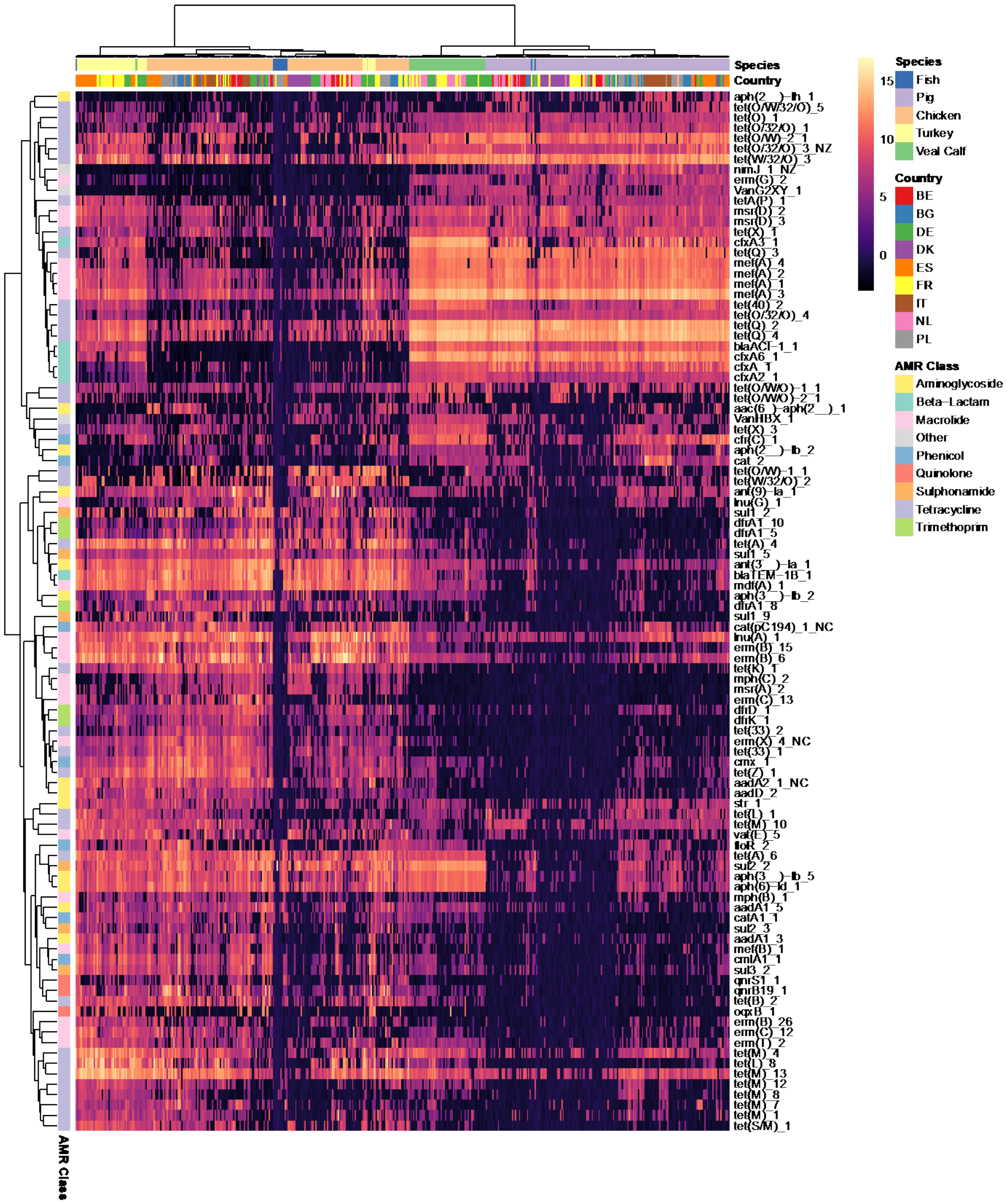

Supp. Fig. 3 | Heat map of AMR gene abundances. CLR-transformed abundances of AMR genes are indicated by the colors of each field. Rows represent AMR genes and columns represent metagenomic samples. Both AMR genes and samples were clustered using Ward linkage clustering. For genes, the clustering was performed on Pearson correlation coefficients. For samples, clustering was based on Euclidean distances. While only the 100 genes with the highest variance in CLR space are displayed here, the presented clustering of samples is based on all 1,032 identified AMR genes.

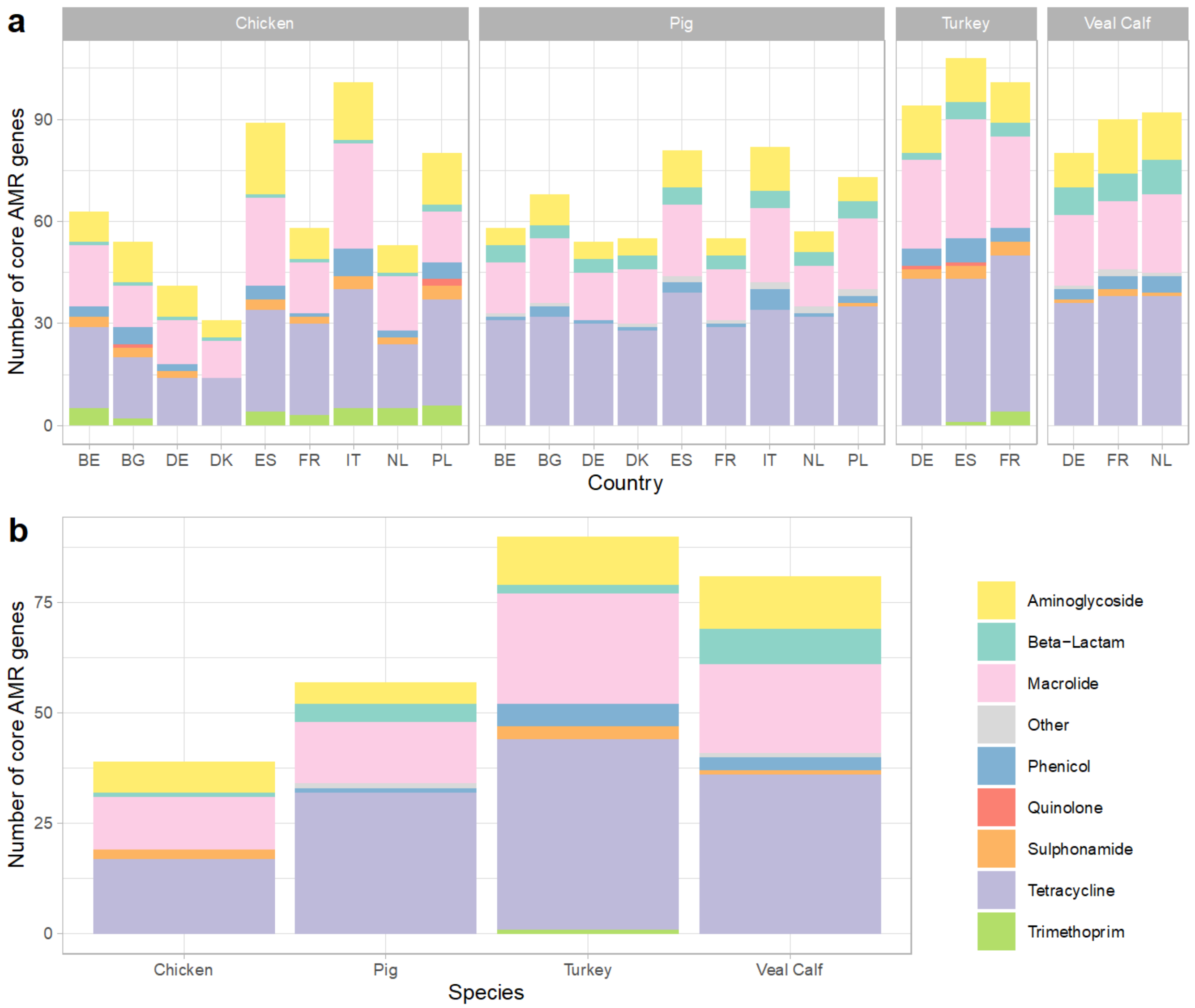

Supp. Fig. 4 | Size of core resistomes. Core AMR genes are genes that appear in at least ca. 90 % of herds in the relevant cohort. **a:** Number of core AMR genes in each country cohort, stratified by host species. Here, core AMR genes are genes that appear in at least eighteen herds from the country cohort. **b:** Number of core AMR genes for each host species. For chicken and pig, core AMR genes are genes that appear in at least 162 herds from this cohort. For turkey and veal calf, the minimum number is 54 herds.

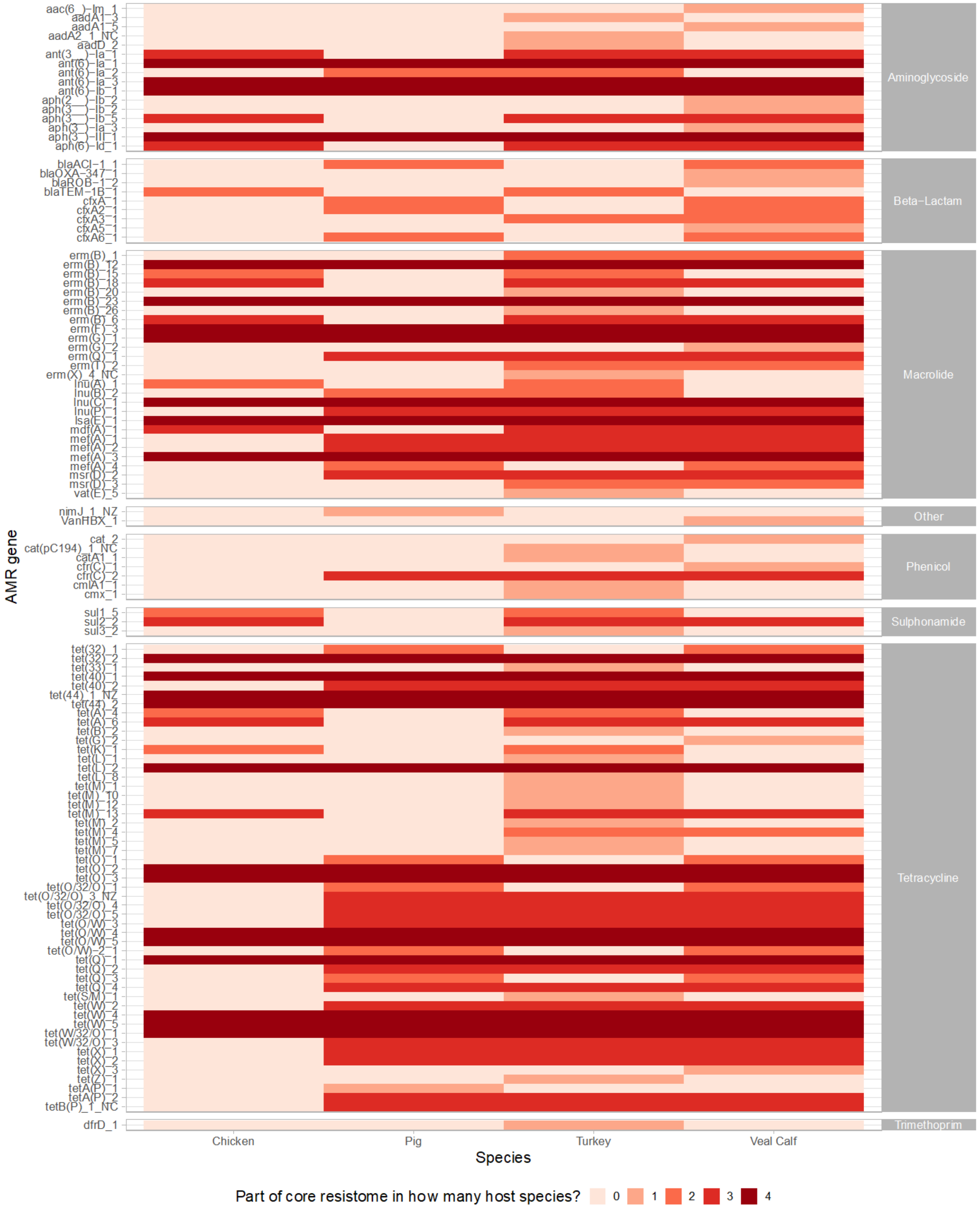

Supp. Fig. 5 | Host species core resistomes. Presence of AMR genes in each species' core resistome is represented in shades of red. Darker shades indicate that the gene is present in a higher number of host species core resistomes. Core AMR genes are genes that appear in at least around 90 % of herds (at least 162 for pig and chicken or at least 54 for veal calf and turkey).

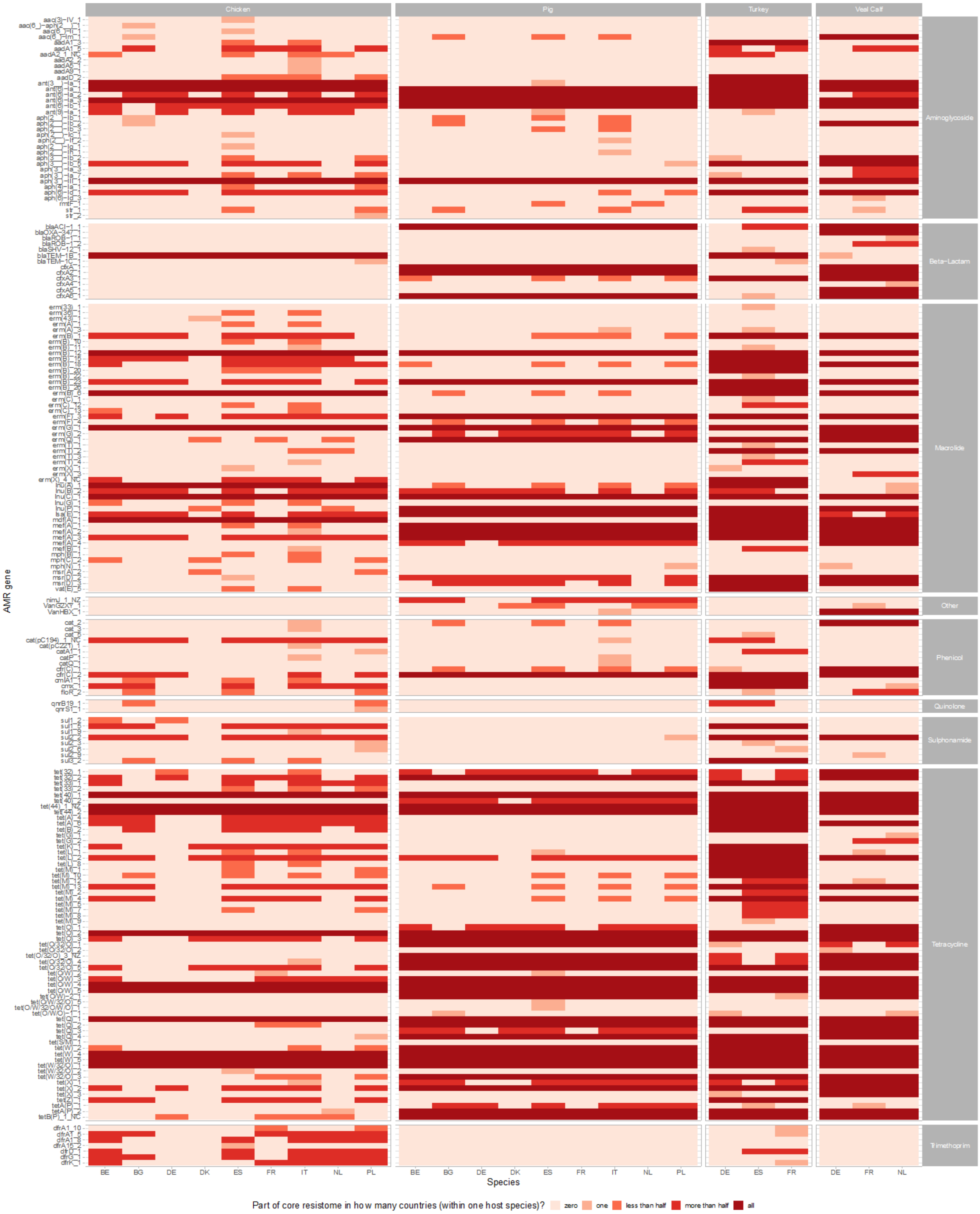

Supp. Fig. 6 | Country cohort core resistomes. Presence of AMR genes in the core resistome of each country cohort is represented in shades of red. Darker shades indicate that the gene is present in a higher number of country core resistomes within the corresponding host species. Core AMR genes are genes that appear in at least eighteen herds

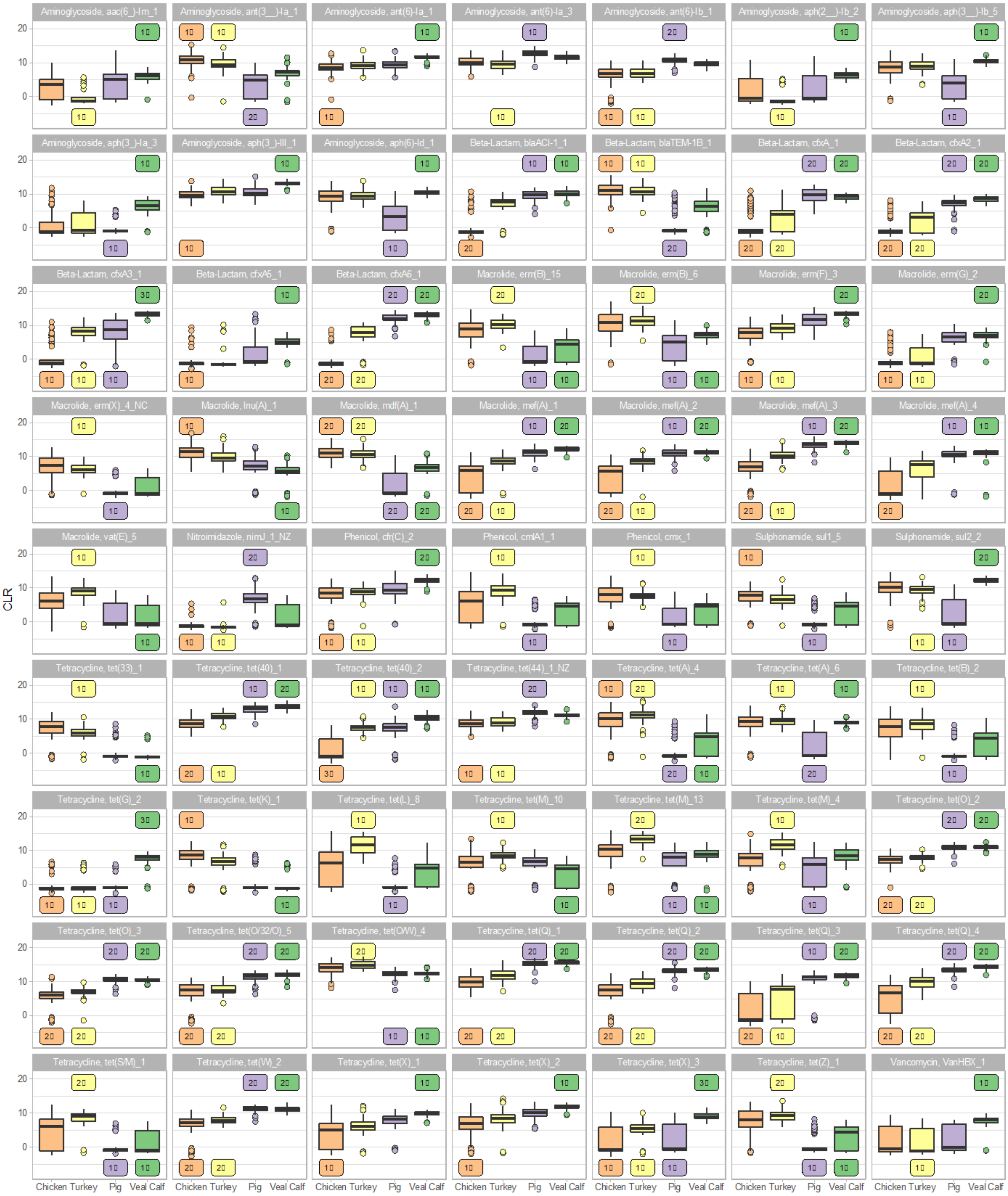

Supp. Fig. 7 | AMR gene abundances and host species effects. CLR value distributions for all AMR genes showing at least one substantial pairwise host species effect (ALDEx2: absolute effect size > 1 & overlap < 0.05). Horizontal box lines represent the first quartile, the median, and the third quartile. Whiskers extend to the smallest and largest data points within the interval [first quartile − 1.5 × interquartile range (IQR), third quartile + 1.5 × IQR]. Data points outside this interval are plotted as circles.

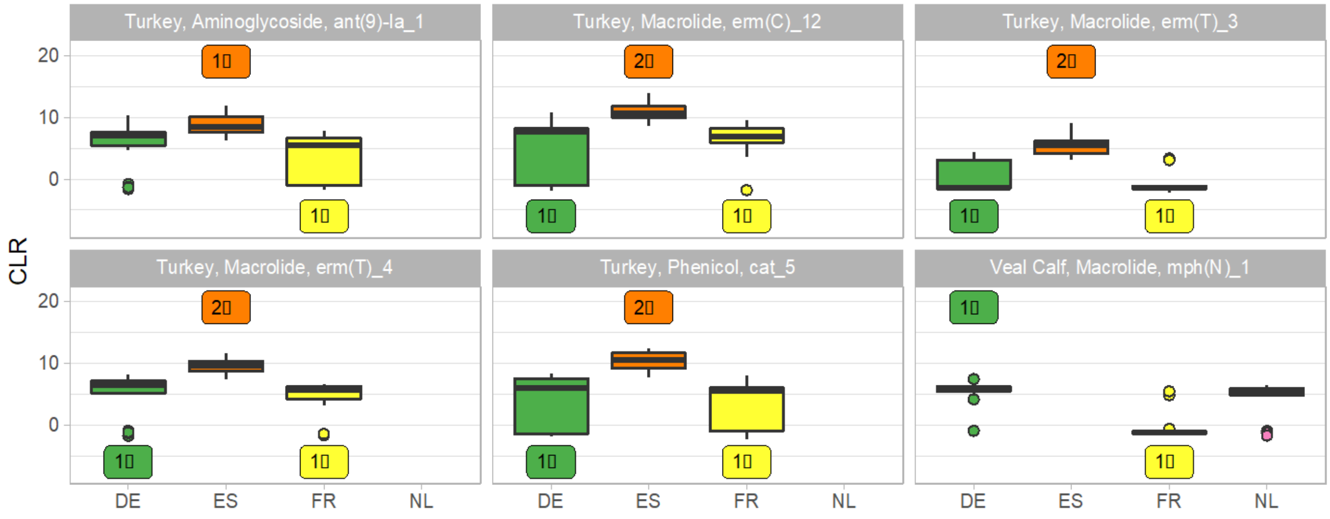

Supp. Fig. 8 | Country effects on AMR gene abundances in herds of turkey and veal calf. CLR value distributions for all AMR genes showing at least one substantial pairwise country effect in herds of turkey or veal calf (ALDEx2: absolute effect size > 1 & overlap < 0.05). Horizontal box lines represent the first quartile, the median, and the third quartile. Whiskers extend to the smallest and largest data points within the interval [first quartile − 1.5 × interquartile range (IQR), third quartile + 1.5 × IQR]. Data points outside this interval are plotted as circles.

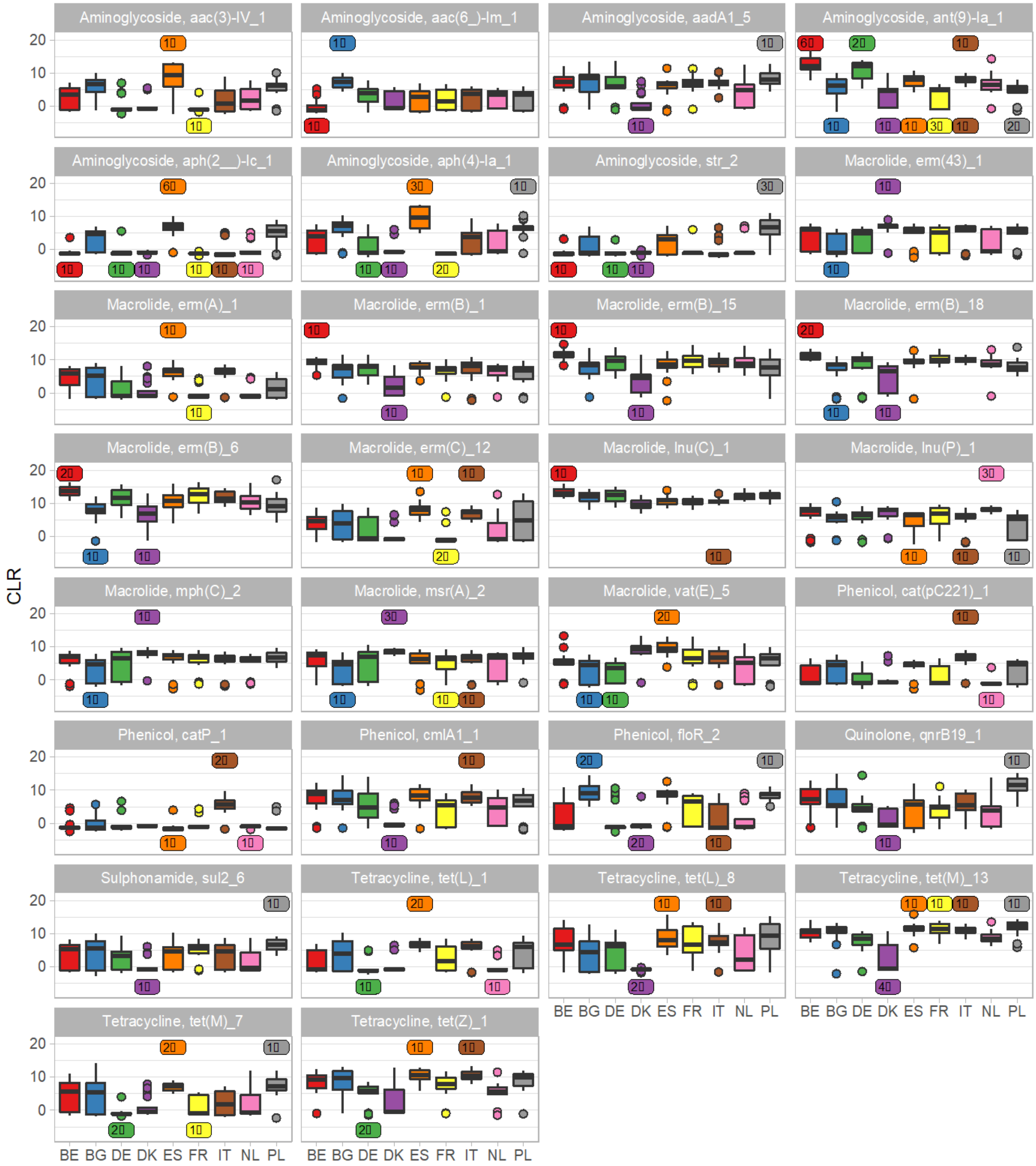

Supp. Fig. 9 | Country effects on AMR gene abundances in chicken herds. CLR value distributions for all AMR genes showing at least one substantial pairwise country effect in chicken herds (ALDEx2: absolute effect size > 1 & overlap < 0.05). Horizontal box lines represent the first quartile, the median, and the third quartile. Whiskers extend to the smallest and largest data points within the interval [first quartile − 1.5 × interquartile range (IQR), third quartile + 1.5 × IQR]. Data points outside this interval are plotted as circles.

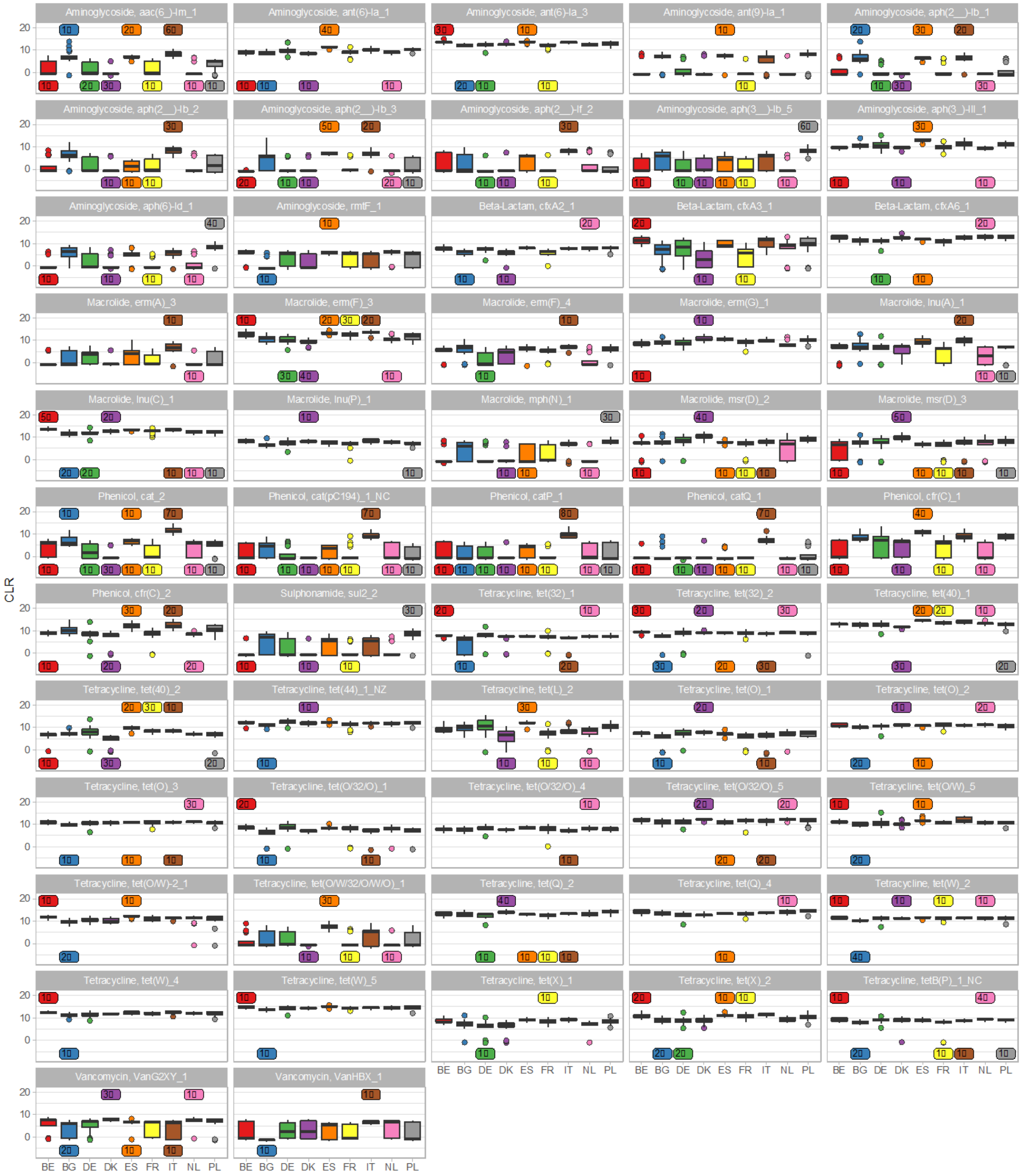

Supp. Fig. 10 | Country effects on AMR gene abundances in pig herds. CLR value distributions for all AMR genes showing at least one substantial pairwise country effect in pig herds (ALDEx2: absolute effect size > 1 & overlap < 0.05). Horizontal box lines represent the first quartile, the median, and the third quartile. Whiskers extend to the smallest and largest data points within the interval [first quartile − 1.5 × interquartile range (IQR), third quartile + 1.5 × IQR]. Data points outside this interval are plotted as circles.

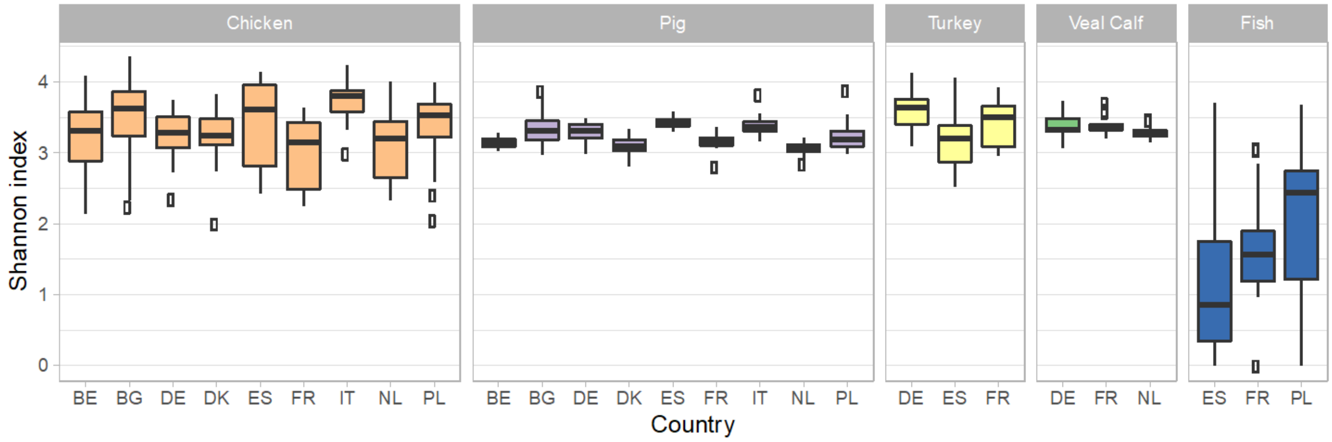

Supp. Fig. 11 | Shannon Evenness of ARGs in cohorts of host species and country. Horizontal box lines represent the first quartile, the median, and the third quartile. Whiskers extend to the smallest and largest data points within the interval [first quartile − 1.5 × interquartile range (IQR), third quartile + 1.5 × IQR]. Data points outside this interval are plotted as circles.
